# Pneumatosis intestinalis after etoposide-based chemotherapy in a patient with metastatic small cell lung cancer: successful conservative management of a rare condition

**DOI:** 10.1590/S1679-45082016RC3597

**Published:** 2016

**Authors:** Luiza Dib Batista Bugiato Faria, Carlos Henrique dos Anjos, Gustavo dos Santos Fernandes, Igor Fernando da Silva Carvalho

**Affiliations:** 1 Hospital Sírio-Libânes, Brasília, DF, Brazil.; 3 Hospital Brasília, Brasília, DF, Brazil.

**Keywords:** Pneumatosis cystoids intestinalis/chemically induced, Etoposide/administration & dosage, Etoposide/adverse effects, Carboplatin/administration & dosage, Carboplatin/adverse effects, Lung neoplasms/drug therapy, Neoplasm metastasis, Case reports

## Abstract

A 69-year-old male patient, smoker, was diagnosed with small cell lung cancer metastatic to lung, liver and central nervous system. He received chemotherapy with carboplatin AUC 5 on day 1 and etoposide 100mg/m^2^ on days 1, 2 and 3. During the first cycle, the patient presented with febrile neutropenia and abdominal distension. Chest, abdomen and pelvis computed tomography scan was performed and detected gas dissecting the wall of sigmoid colon extending to the mesosigmoid. Patient had no abdominal pain, nausea, vomiting, and on physical examination he had no peritoneal irritation, tachycardia or hemodynamic instability compatible with perforation or acute abdomen. Therefore, the radiological finding was interpreted as pneumatosis intestinalis caused by chemotherapy with etoposide. Pneumatosis resolved after continuous oxygen therapy. The second cycle was administered after a complete resolution of the clinical condition and etoposide dose was reduced by 30%. The patient experienced a remarkable evolution.

## RESUMO

Paciente do gênero masculino, 69 anos, fumante, diagnosticado com câncer de pulmão de pequenas células, metastático para pulmão, fígado e sistema nervoso central. Foi administrada quimioterapia com carboplatina AUC 5 no dia 1 e etoposídeo 100mg/m^2^ nos dias 1, 2 e 3. Durante o primeiro ciclo, o paciente apresentou neutropenia febril e distensão abdominal. Tomografias de tórax, abdome e pelve detectaram gás dissecando a parede do cólon sigmoide, com extensão para o mesossigmoide. O paciente não apresentava dor abdominal, náusea, vômito e não tinha sinais de irritação peritoneal, taquicardia ou instabilidade hemodinâmica compatíveis com perfuração ou abdome agudo. O achado radiológico foi interpretado como pneumatose intestinal causada por etoposídeo. A resolução do quadro ocorreu após suplementação de oxigênio. O segundo ciclo foi administrado após resolução completa do quadro, com redução da dose do quimioterápico em 30%. O paciente evoluiu de forma bastante satisfatória.

## INTRODUCTION

Pneumatosis intestinalis (PI) is an uncommon clinical sign, and consists of the existence of gas in the wall of the gastrointestinal tract; it can occur in children and adults.^(1)^ Duvernoy first described this condition in a pathology study,^(2)^ in 1973, and since then, several studies and case reports have been published, with an estimated incidence of 0.03%.^(3)^ Due to the emerging medical imaging techniques, specially computerized tomography (CT) scan, the incidence of PI has increased. It is important to mention that PI is not commonly associated with peritonitis and it is a self-limiting condition in most cases.^(4)^ The clinical conditions most associated with PI are immunosuppression and the increased permeability of the gastrointestinal mucosa in patients undergoing chemotherapy.^(5)^ Although a rare condition, it should be carefully interpreted and requires an appropriate investigation to make appropriate differential diagnosis.^(5)^

## CASE REPORT

A 69-year-old male patient, smoker and with a previous diagnosis of chronic obstructive pulmonary disease (COPD), was admitted to the emergency room with mental confusion and ataxia. The patient was in a good clinical condition, and there were no signs suggesting any source of respiratory, urinary or central nervous system infection. Chest X-ray and magnetic resonance imaging (MRI) of the brain were requested.

Chest X-ray revealed a dense nodule of approximately 13mm in diameter, in the periphery of the left lower lobe of the lung, and a prominent left hilar lymph node. The MRI detected multiple metastases associated with vasogenic edema.

According to clinical history and radiologic findings, the hypothesis of small cell lung cancer was made. Chest and abdominal CT was performed to determine the stage of disease and track other disease sites. The abdomen image revealed hepatic metastasis, unspecific nodular thickening of adrenal glands, retroperitoneal lymphadenopathy and a slightly enlarged prostate. The chest image showed a left hilar mass with mediastinal invasion, with possible invasion of the descending aorta and occlusion of the left inferior pulmonary vein, besides a small pulmonary nodule to the right. Biopsy of liver metastasis was performed and confirmed diagnosis as small cell carcinoma. Immunohistochemistry result was consistent with the hypothesis of small cell lung cancer.

**Figure 1:**
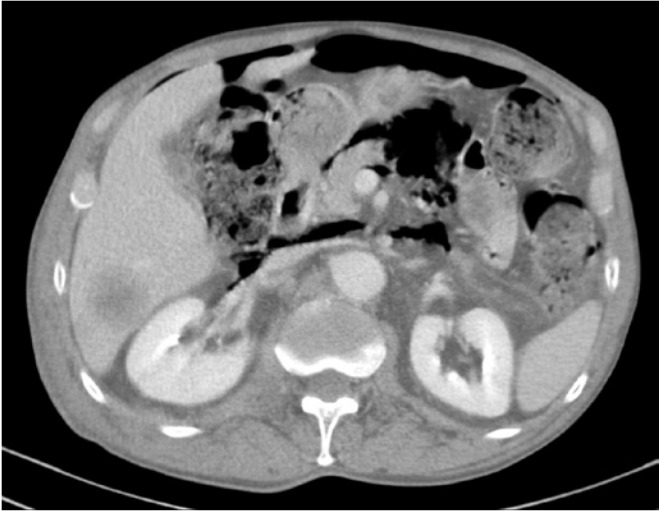
Abdominal computed tomography shows pneumatosis intestinalis

First line palliative chemotherapy with carboplatin AUC 5 on day 1 and etoposide 100mg/m^2^ on days 1 to 3, every 21 days was administered. On day 8 of the first cycle, the patient presented with abdominal distention and fever. Complete blood count showed neutropenia. The patient received antibiotics and CT scans of the chest, abdomen, pelvis and sinuses were performed. Comparing with the initial scans, these images showed presence of gas dissecting the wall of sigmoid colon extending to the mesosigmoid, retroperitoneum and posterior mediastinum, and a bulky pneumoperitoneum without gas in the portal venous system. Moreover, there was the additional finding of a hematoma in the psoas major muscle associated with edema in the adjacent subcutaneous tissue (Figure 1).

**Figure 2:**
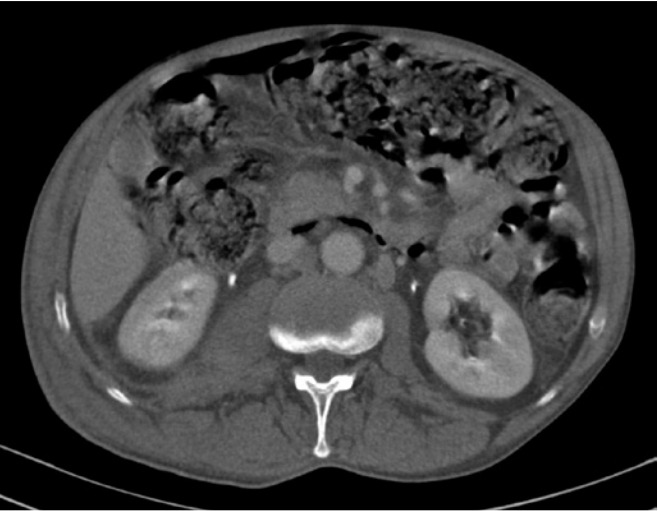
Another computed tomography slice showing pneumatosis intestinalis

The hepatic lesions, nodular thickening of the adrenal gland and retroperitoneal lymphadenopathy had reduced size. The chest CT scan revealed a small pneumomediastinum and a significant reduction of the left hilar mass. The patient did not complain of abdominal pain, nausea, vomiting, and had no clinical signs compatible with peritoneal irritation or acute abdomen perforation. After the presumptive diagnosis of PI, the patient was given continuous high dose of oxygen and maintained antibiotic regimen. The abdominal distension progressively improved and 2 weeks after oxygen therapy new scans showed resolution of pneumatosis image according to figure 2. The etoposide dose was reduced by 30% in the second cycle of treatment. There were no new signs of PI after dose reduction. After four cycles of chemotherapy, the patient presented a partial response (reduction of mediastinal lymph nodes and hepatic lesions). Afterwards, the patient was referred to whole brain radiation therapy.

## DISCUSSION

Pneumatosis intestinalis is a rare condition, and its pathophysiology is still poorly understood. Three main theories have been proposed for the pathogenesis of this condition.^(6)^ The mechanical theory postulates that the condition develops as a result of increased intraluminal pressure which allows gas to infiltrate the bowel wall via mucosal defects. In contrast, the bacterial theory postulates that PI occurs when the submucosal localization of fermenting bacteria (*e.g*., *Clostridium difficile* and *Escherichia coli*) leads to the production of gas that subsequently accumulates in the submucosa. In addition, the pulmonary theory suggests that gas released by rupture of alveoli travels through the mediastinum and retroperitoneum into the bowel wall.

The clinical manifestations of PI range from incidental findings to life-threatening complications, such as bowel ischemia. The management can be conservative in many patients.

Many agents have been reported to be associated with PI, including methotrexate, fluorouracil, paclitaxel, docetaxel, intravenous etoposide, vascular endothelial growth factor inhibitors (VEGF-A), such as bevacizumab, and tyrosine kinase inhibitors (TKIs), like sorafenib and sunitinib.^(7)^

In the present case, the pulmonary disease may have contributed to the development of the PI. The patient presented with a stable clinical condition and a conservative management was appropriate and efficient. Since there were no reports on the literature regarding carboplatin and the development of this condition, and the patient had not taken any other medicine, etoposide was considered the cause of PI.

We concluded that although a rare complication, it should be remembered that patients on chemotherapy might develop PI. The clinical presentation must be carefully evaluated to decide whether surgery or conservative management is suitable.
